# Supplementary material for: Toward sustainable community-based tourism development: Perspectives from local people in Nhon Ly coastal community, Binh Dinh province, Vietnam
Source: PLoS One. 2023 Oct 31;18(10):e0287522. doi: 10.1371/journal.pone.0287522 (PMC10617687; doi:10.1371/journal.pone.0287522)
Supplement: Attachment — Submitted filename: Fiji Paper review.docx [file pone.0287522.s001.docx]

**Manuscript Number: PONE-D-22-32881**

**Manuscript Title: Toward sustainable community-based tourism development: Perspectives from local people in Nhon Ly coastal community, Binh Dinh Province, Vietnam**

**This is a good research article**

I have reviewed the above research articles:

**The article cannot be published in its present form**. My suggestions are as follows:

a)    There are grammatical mistakes and also problems in tense. A few I am mentioning: in 138 number line **believe** has been used should be **reveal**

b)    Line 169 **UNWTO** full form should be used

c)    176 instead of **currently presently** should be used.

d)    In **270 Km2 is not correct full should be used**

e)    In many places **km has been used but in full form and must be used**

f)     In 315 line it should be **research methodology**

g)    **Also sampling may be used in this area**

**h) During study time, Participant Observation was used now a days new Research Methodology is known as PRA (Participatory Rural Apprisal), not Participant Observation used as a study.**

**h)**   In 319, Primary Data **conducted should be collected**

**i)**      In Table -2 it should be **respondents**

**j)**     Table-3  and Table -4 are not correct  it should be mentioned **as Chart -3, chart-4**

**k)**   In the article **some, many words** have been used which should be avoided. It can be mentioned as …...**around % or number approx.**

**l)**      **In the FINDINNS Present and Past tenses have been mixed. All MAINLY Should be in the PAST tense. FINDINGS SHOULD BE CORRECTED**

--

**Dr. Shankar Chatterjee**

**Former Professor & Head (CPME),NIRD & PR (Govt. of India), Hyderabad,  India**
