# Supplementary material for: Toward sustainable community-based tourism development: Perspectives from local people in Nhon Ly coastal community, Binh Dinh province, Vietnam
Source: PLoS One. 2023 Oct 31;18(10):e0287522. doi: 10.1371/journal.pone.0287522 (PMC10617687; doi:10.1371/journal.pone.0287522)
Supplement: Attachment — Submitted filename: Response to reviewers.docx [file pone.0287522.s002.docx]

Dear Prof. Sakul Kundra,

PhD in History
Academic Editor
PLOS ONE

Thank you for the opportunity to resubmit the manuscript. We have carefully reviewed the comments, suggestions, and questions from the reviewer. First and foremost, we would like to express our deep gratitude to you and the reviewers for providing constructive feedback and encouraging the publication of our manuscript. We have taken all of the concerns, questions, and corrections from the reviewers into full consideration to improve the manuscript. Please find our detailed responses to the reviewers’ comments below. We have also submitted the revised manuscript with track changes attached to this letter. Wishing you all the best!

Sincerely,

On behalf of the authors

Dr. Nguyen Van Hoang

**Journal requirements:**

When submitting your revision, we need you to address these additional requirements.

1. Please ensure that your manuscript meets PLOS ONE's style requirements, including those for file naming. The PLOS ONE style templates can be found at

<https://journals.plos.org/plosone/s/file?id=wjVg/PLOSOne_formatting_sample_main_body.pdf> and <https://journals.plos.org/plosone/s/file?id=ba62/PLOSOne_formatting_sample_title_authors_affiliations.pdf>.

*We have checked the PLOS ONE style templates and followed the instruction accordingly.*

2. Your ethics statement should only appear in the Methods section of your manuscript. If your ethics statement is written in any section besides the Methods, please move it to the Methods section and delete it from any other section. Please ensure that your ethics statement is included in your manuscript, as the ethics statement entered into the online submission form will not be published alongside your manuscript.

*We have included the ethics statement in the Methods section of our manuscript only.*

3. We note that [Figure 1] in your submission contain [map/satellite] images which may be copyrighted. All PLOS content is published under the Creative Commons Attribution License (CC BY 4.0), which means that the manuscript, images, and Supporting Information files will be freely available online, and any third party is permitted to access, download, copy, distribute, and use these materials in any way, even commercially, with proper attribution. For these reasons, we cannot publish previously copyrighted maps or satellite images created using proprietary data, such as Google software (Google Maps, Street View, and Earth). For more information, see our copyright guidelines: <http://journals.plos.org/plosone/s/licenses-and-copyright>.

We require you to either (1) present written permission from the copyright holder to publish these figures specifically under the CC BY 4.0 license, or (2) remove the figures from your submission:

*We have removed Figure 1 and replaced it with another figure taken by the author.*

4. We note that Figures 3 and 4 includes an image of a [patient / participant / in the study].

*We have removed Figure 3 and 4 from our manuscript*

5. Please review your reference list to ensure that it is complete and correct. If you have cited papers that have been retracted, please include the rationale for doing so in the manuscript text, or remove these references and replace them with relevant current references. Any changes to the reference list should be mentioned in the rebuttal letter that accompanies your revised manuscript. If you need to cite a retracted article, indicate the article’s retracted status in the References list and also include a citation and full reference for the retraction notice.

*We have reviewed our reference list according to the guideline.*

**Reviewers’ comments**

| **Reviewer 2’s comments** | **Authors’ responses** |
| --- | --- |
| a) There are grammatical mistakes and also problems in tense. A few I am mentioning: in 138 number line believe has been used should be reveal | Thank you very much for your comments. We have corrected our manuscript based on your suggestions. Please see details in line 143 on page 7. |
| b) Line 169 UNWTO full form should be used | Thank you very much for your comments. We have corrected our manuscript based on your suggestions. Please see details in line 176 on page 8. |
| c) 176 instead of currently presently should be used. | Thank you very much for your comments. We have corrected our manuscript based on your suggestions. Please see details in line 186 on page 9. |
| d) In 270 Km2 is not correct full should be used | Thank you very much for your feedback. We have made the necessary corrections to our manuscript based on your suggestions. Throughout the paper, we have written “square kilometers” instead of “Km2” |
| e) In many places km has been used but in full form and must be used | Thank you very much for your feedback. We have made the necessary corrections to our manuscript based on your suggestions. Throughout the paper, we have written “kilometers” instead of “Km” |
| f) In 315 line it should be research methodology | Thank you very much for your comments. We have corrected our manuscript based on your suggestions. Please see details in line 335 on page 15. |
| g) Also sampling may be used in this area | Thank you for your comments. We appreciate your feedback but would like to clarify that we may not have fully understood this particular point. Our data was collected primarily through semi-structured interviews, and therefore we did not include the sampling method typically required for questionnaire surveys. |
| h) In 319, Primary Data conducted should be collected | Thank you very much for your comments. We have corrected our manuscript based on your suggestions. Please see details in line 339 on page 15. |
| i) In Table -2 it should be respondents | Thank you for your comment. We have corrected the table 2 based on your suggestions. |
| j) Table-3 and Table -4 are not correct it should be mentioned as Chart -3, chart-4 | Thank you for your feedback. We appreciate your suggestions, but we have decided to use Tables 3 and 4 as they provide supportive information in a tabular format. |
| k) In the article some, many words have been used which should be avoided | Thank you very much for your comments. We have corrected our manuscript based on your suggestions. |
| l) In the FINDINNS Present and Past tenses have been mixed. All MAINLY Should be in the PAST tense. FINDINGS SHOULD BE CORRECTED | Thank you for your comments. We are pleased to inform you that we have incorporated your suggestions into our manuscript, particularly in the Findings section. |
